# ST-deconv: an accurate deconvolution approach for spatial transcriptome data utilizing self-encoding and contrastive learning

**DOI:** 10.1093/nargab/lqaf109

**Published:** 2025-08-27

**Authors:** Shurui Dai, Jiawei Li, Zhiliang Xia, Jingfeng Ou, Yan Guo, Limin Jiang, Jijun Tang

**Affiliations:** Shenzhen Institute of Advanced Technology, Chinese Academy of Sciences, 518005 Guangdong, China; Shenzhen Institute of Advanced Technology, Chinese Academy of Sciences, 518005 Guangdong, China; Shenzhen Institute of Advanced Technology, Chinese Academy of Sciences, 518005 Guangdong, China; Shenzhen Institute of Advanced Technology, Chinese Academy of Sciences, 518005 Guangdong, China; Department of Public Health Sciences, University of Miami, Miami, FL 33136, United States; Department of Public Health Sciences, University of Miami, Miami, FL 33136, United States; Shenzhen Institute of Advanced Technology, Chinese Academy of Sciences, 518005 Guangdong, China

## Abstract

Single-cell RNA sequencing (scRNA-seq) has significantly deepened our understanding of cellular heterogeneity and cell type interactions, providing insights into how cell populations adapt to environmental variability. However, its lack of spatial context limits intercellular analysis. Similarly, existing spatial transcriptomics (ST) data often lack single-cell resolution, restricting cellular mapping. To address these limitations, we introduce ST-deconv, a deep learning-based deconvolution model that integrates spatial information. ST-deconv leverages contrastive learning to enhance the spatial representation of adjacent spots, improving spatial relationship inference. It also employs domain-adversarial networks to improve generalization and deconvolution across diverse datasets. Moreover, ST-deconv can generate large-scale, high-resolution spatial transcriptomic data with cell type labels from single-cell input, facilitating the learning of spatial cell type composition. In benchmarking experiments, ST-deconv outperforms traditional methods, reducing the root mean square error (RMSE) by 13% to 60%, with an RMSE as low as 0.03 for high spatial correlation datasets and 0.07 for low spatial correlation datasets across different transcriptomic contexts. Reconstructing real tissue structure, a purity of 0.68 on mouse olfactory bulb (MOB) and a cell type correlation of 0.76 on human pancreatic ductal adenocarcinoma (PDAC) were achieved. These advancements make ST-deconv a powerful tool for enhancing spatial transcriptomics and downstream analyses of intercellular interactions.

## Introduction

Single-cell RNA sequencing (scRNA-seq) has enabled transcriptome analysis at the single-cell level [1], offering significant insights [2] into cellular heterogeneity and fundamental biological processes, such as early embryonic development [3]. Despite its success in uncovering cellular diversity [4], developmental pathways, and disease mechanisms, and even its ability to map the generated data to the tissue of origin [5], scRNA-seq is constrained by the lack of spatial context. This limitation hampers the accurate interpretation of intercellular relationships and impairs the construction of tissue models [6] that are essential for downstream applications [7], such as the design of cell factories [8]. Advances in spatial transcriptomics have enabled the study of tissue cell type architecture and cell–cell interactions [9], laying a foundation for a deeper understanding of tissue structure [10–12] and function.

Third-generation spatial transcriptomics addresses this gap by preserving spatial information during sequencing. Technologies such as MERFISH [13] allow for near-genome-wide spatially resolved RNA analysis at the single-cell level, identifying RNA species within specific subcellular compartments, linking transcriptional states to distinct cell cycle phases, and revealing spatial patterns of transcriptional activity [14]. However, no single spatial transcriptome technology is optimal for all use cases, and inherent trade-offs, along with implementation challenges, have restricted the large-scale adoption of some techniques. The most commonly used techniques—such as Slide-seq [15, 16], 10× Genomics Visium [17], laser capture microdissection (LCM) [18, 19], GeoMX Digital Spatial Profiling (DSP) [20], and Tomo-seq [21]—still suffer from limitations, particularly in spatial resolution [9].

Low-resolution spatial transcriptome data, due to experimental constraints and technical limitations, often fail to capture precise cell locations and boundaries [17]. This lack of resolution presents challenges for accurate cell type identification and spatial analyses [22], limiting our understanding of tissue architecture and cellular function. Although newer, high-throughput single-cell transcriptomes [11] have become available, a considerable amount of valuable low-resolution spatial transcriptomic data remains [23], presenting an economic and research opportunity.

The main strategies for spatial transcriptomic analysis include clustering [24, 25], deconvolution [26], feature selection [27, 28], ranking [29], and association studies [30]. The position of a cell relative to its neighbors and non-cellular structures can provide valuable insights into defining cell phenotypes, cell states, and ultimately the functions of both cells and tissues [31]. A key task in this context is to improve the spatial resolution of existing data through deconvolution techniques, which aim to resolve ambiguities in cell positioning. Traditional machine learning models have been widely applied to address this challenge, offering solutions for improving the accuracy of spatial analyses in low-resolution datasets.

Over the past four years, several computational methods, such as the BayesSpace algorithm, have leveraged the neighborhood structure in spatial transcriptomic data to enhance subspot-level resolution, achieving super-resolution image analysis by utilizing Bayesian statistics [32], and several computational methods have been developed to enhance the resolution of spatial transcriptomic analyses. These methods fall into two primary categories.

- Traditional machine learning-based deconvolution. Robust cell type decomposition (RCTD) [33] employs a traditional log-linear model, integrating the Berthon factor analysis to account for spatial bias and normalization across different platforms, resulting in a highly robust deconvolution model. Similarly, Stereoscope [34] uses a negative binomial distribution, considering spatial and platform variables to achieve high-precision deconvolution. cell2location [35], a Bayesian model based on a negative binomial distribution, leverages prior information on cell abundance across spatial locations to generate fine-grained spatial transcriptomic data. spatialDWLS [36] combines gene set enrichment analysis [parametric analysis of gene set enrichment (PAGE)] with weighted least squares to infer cell types. Although integrating spatial and histological data can enhance accuracy for some datasets, the improvement is not always substantial when compared solely with gene expression data [37]. SPOTlight [38] utilizes non-negative matrix factorization (NMF) to extract feature vectors, followed by non-negative least squares fitting to determine the optimal predictive expression. CARD [39], another model employing NMF, optimizes the use of spatial information, outperforming previous methods. Despite these advancements, there is still room for improvement in the performance metrics of existing approaches. Given the successes of deep learning in numerous applications, there is strong potential for deep learning to excel in spatial transcriptomic deconvolution tasks by improving spatial information extraction and feature selection.
- Deep learning-based deconvolution. DeepST is a powerful deep learning framework that excels in identifying spatial domains, outperforming state-of-the-art methods on human dorsolateral prefrontal cortex benchmark datasets. Additionally, it dissects spatial domains in breast cancer tissues at a finer scale, demonstrating its accuracy and versatility [40]. The CellDART model, a domain-adversarial approach for classifying cell types in spatial transcriptomics, was benchmarked against several methods, including cell2location, SPOTlight, DSTG, Scanorama, RCTD, and Seurat, across various biological tissue sections. The results showed that CellDART outperformed these methods in terms of area under the curve (AUC) values for cell type deconvolution, confirming its superior effectiveness. Similarly, GraphST [41], designed to infer cellular locations within spatial transcriptomic data, exhibited better performance than cell2location but faced challenges in spatial interpretability compared with traditional machine learning models. This limitation in spatial information utilization highlights the need for a more sophisticated approach. Previous methods struggle with embedding spatial information accurately or fail to capture the intricate spatial relationships between cells. Traditional machine learning methods often underperform in representing spatial context, resulting in suboptimal deconvolution outcomes. Moreover, many of these methods rely on simplified models that do not fully leverage the rich spatial and histological data, leading to limitations in precision and interpretability [37]. Addressing these challenges requires more advanced models that can better extract and integrate spatial features.

To address these shortcomings, we propose incorporating contrastive learning (CL) into deconvolution frameworks to better integrate spatial information. CL has been widely adopted in representation learning tasks across various domains, such as computer vision and natural language processing, due to its ability to improve feature discrimination by leveraging instance-level comparisons. In spatial transcriptomics, where spatial proximity holds significant biological meaning, applying CL can help distinguish subtle differences in cell-type-specific expression profiles. CL distinguishes between positive and negative sample pairs based on spatial proximity, thereby enhancing the model’s ability to capture and utilize spatial relationships effectively. Specifically, positive groups are constructed from spatially adjacent regions that are expected to share similar transcriptomic profiles, whereas negative groups are drawn from distant regions with potentially distinct cell type compositions. This contrastive setup ensures that the model learns to emphasize biologically relevant spatial features while reducing the influence of noise and batch effects. Furthermore, we employ the domain-adversarial neural network (DANN), a widely used technique in domain adaptation that leverages adversarial learning to reduce domain shifts between different data distributions. Originally developed for applications such as sentiment analysis and image classification, DANN has also shown promise in biomedical domains by aligning feature distributions across datasets, thereby enhancing model robustness. As part of the training process, an adversarial component mitigates the noise introduced by simulated spatial data with cell type labels, thus improving the overall generalization capability of the deconvolution model. In the context of spatial transcriptomics, variations in experimental conditions and noise from sequencing technologies can introduce systematic biases. By integrating DANN, ST-deconv learns a domain-invariant feature space, enabling it to generalize across datasets while preserving biologically meaningful spatial structures. Together, ST-deconv represents a significant step forward in the accurate deconvolution of spatial transcriptomes, allowing for more precise cell type classification and spatial relationship inference across various biological contexts. By incorporating CL and DANN, ST-deconv not only refines cell type deconvolution but also enhances its resilience to dataset variations, making it a powerful tool for spatial transcriptomic analysis in diverse biological and clinical settings.

## Materials and methods

**Figure 1.**
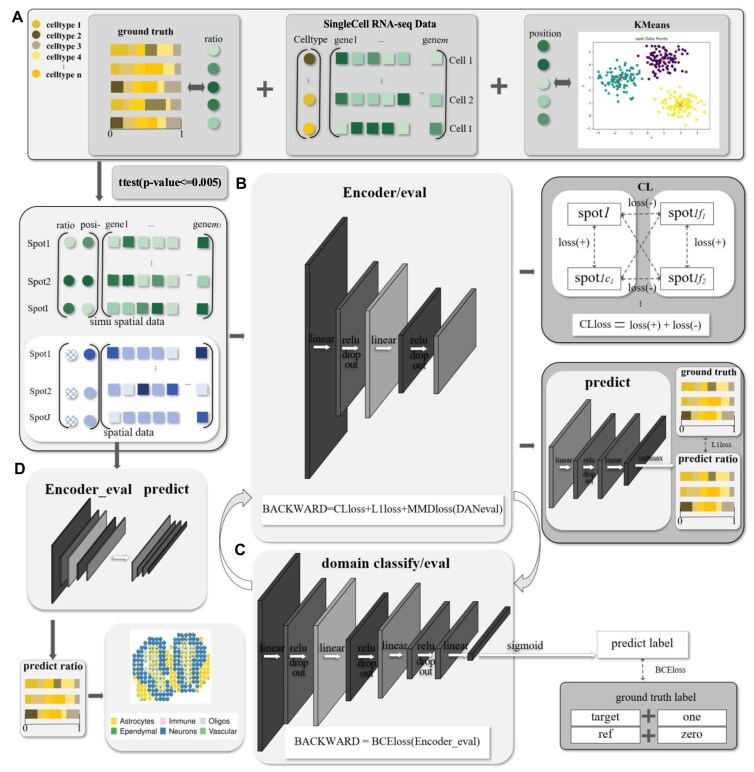
An overview of ST-deconv. (**A**) Data pre-processing consists of three main steps within ST-deconv: simulating spatial transcriptome data; generating accurate ratio tags; and producing spatial transcriptome data by integrating these tags with single-cell transcriptome data. Position information labels are assigned based on the similarity of gene expression across spots (spatially defined sequencing spots within a biological tissue) in the spatial transcriptome data. A *t*-test is then applied to identify genes which are differentially expressed, aligning these processed spatial transcriptome data with real spatial transcriptome profiles for further analysis. (**B**) For encoder training, the pre-processed data are input into encoder to extract features, the CLloss of CL module, L1loss of predict module, and BCEloss of domain classify module of eval mode are included in the training of Encoder. (**C**) For adversarial training, the features are extracted by encoder of eval mode and input into the domain classify module to calculate BCEloss. (**D**) Testing with real spatial transcriptome data. Following a *t*-test, data are input into an encoder to extract features for BCE loss calculation in the domain classification module, facilitating refined analysis and validation.

For the task of deconvoluting spatial transcriptomes with low precision, we introduce ST-deconv, a novel cell type deconvolution model that leverages CL to fully utilize spatial transcriptomic information while incorporating domain adversarial techniques to enhance model generalization across datasets. The ST-deconv model consists of three key components (Fig. 1): data generation; embedding model; and prediction module. This modules ensures effective spatial feature extraction and robust performance across various spatial transcriptomic contexts.

### Generation and pre-processing of simulated spatial transcriptome data

#### Simulating spatial transcriptome data with cell type proportions

Due to the lack of cell type labels in ground truth spatial transcriptome data and the absence of spatial location information in single-cell transcriptome data, we developed a randomized approach to generate ground truth data that reflect cell type proportions within spatial transcriptomes. This method determines the number of single-cell samples for each spatial transcriptome spot by leveraging the multiplicity inherent in both spatial and single-cell transcriptome data from comparable tissue types, assuming minimal variability in cell counts across similar tissues. By combining the total number of single-cell samples and the proportion of cell types for each spatial transcriptome spot, we calculate the expression values for each cell type within the spatial transcriptome spot and sum these values to obtain the overall expression value for each spatial transcriptome spot. Three-dimensional spatial transcriptome technologies provide insights into intercellular relationships by preserving spatial context. For our simulated spatial transcriptome data, we applied k-means clustering to identify groups of similar data points, optimizing their coordinates to reflect spatial proximity, which often correlates with expression similarity. By generating spatial coordinates based on cell type proportions, we created spatial transcriptome data that reflect realistic spatial distributions. This diversity in random generation enhances the model’s learning of associations between spatial site characteristics and cell type composition. However, the generated data are not actual spatial transcriptome data and still exhibit certain limitations in authenticity. Additionally, no extra noise was introduced during the generation process.

#### Integrating simulated and real data using CARD for improved spatial fidelity

Simple random generation may not fully capture the spatial complexities needed for accurate transcriptome deconvolution. To address this, we used the CARD method [39], which incorporates morphological information from real samples to simulate data with higher authenticity. CARD preserves hierarchical spatial patterns found in frozen tissue samples, offering a realistic basis for simulations. Specifically, simulations for mouse olfactory bulb (MOB) leveraged its circular structure to validate the generated data against actual spatial transcriptome data. Our ST-deconv model was adapted to integrate simulated data with real spatial transcriptome datasets, using the union of gene lists from both sources to identify and extract differentially expressed genes for spatial transcriptome data. The simulated data may be model generated or externally sourced, and this union with spatial transcriptome data prepares the model for domain adversarial learning. For example, simulated spatial transcriptome data generated using the CARD method, alongside corresponding real spatial transcriptome data, were both utilized in model training to enhance robustness and accuracy.

#### Enhancing simulated spatial transcriptome data for robust training

In high-dimensional data analysis, such as transcriptomic data with an extensive number of genes, selecting genes with significant mean differences enables effective dimensionality reduction, retaining highly informative gene features and thus improving the computational efficiency and predictive accuracy of the ST-deconv model. In ST-deconv, differentially expressed genes were selected using a *t*-test, improving training efficiency by filtering with a *P*-value threshold of 0.005. After filtering, 13 064 genes were retained for model training. This gene selection criterion significantly enhances data quality, supporting more accurate model training.

### High-generality embedding for spatial transcriptome data

#### Gene expression embedding

The ST-deconv model is structured around an encoder architecture. Each spatial transcriptome locus in the input data matrix is represented as a concatenated vector, which integrates gene expression data with its corresponding two-dimensional spatial coordinates. During training, the pre-processed data are input into the encoder to generate embeddings for both simulated and real spatial transcriptomes. The input dimensions of the encoder correspond to the number of genes selected by *t*-tests following pre-processing. The encoder consists of two layers, each utilizing a linear transformation followed by a ReLU activation function. In the first layer, the input dimensions are reduced from the original size to 512. A dropout rate of 0 is applied to values that fall below the ReLU threshold. The second layer further reduces the dimensions from 512 to 256, with a dropout rate of 0.5 applied after the ReLU activation. The final layer again reduces the dimensions from 256 to a specified size, implementing a dropout rate of 0.3. The model was trained with a learning rate of 0.0003, which controls the speed of parameter updates. A learning rate that is too high may lead to instability during training, while one that is too low can slow down convergence. A batch size of 32 was used, representing the number of samples processed in each training step, which affects both computational efficiency and model generalization. In experiments involving adversarial training, this combination of learning rate and batch size achieved a good balance between efficiency and training stability.

#### Spatial information embedding based on contrastive learning

CL is a self-supervised learning technique [42] that aims to extract valuable information from data by creating a task embedding different augmentations of the same data sample closer together while pushing embeddings from different samples apart, thereby optimizing feature representation [43]. In this approach, two similar samples are called a positive pair, whereas two dissimilar samples form a negative pair.

Given that our inverse convolution task lacks data on the proportion of cell types at specific spatial spots, we leverage CL to enable the model to learn meaningful feature representations without labeled data. This approach is grounded in the premise that spatially similar cells will exhibit more similar features. By creating positive and negative sample pairs based on spatial proximity, we maximize the utilization of spatial information to differentiate between various spots.

In the ST-deconv model, CL distinguishes embeddings generated by the encoder into positive and negative sample spots based on their spatial locations. To accurately differentiate these sample spots, we apply a sampling strategy that aligns with the biomorphological characteristics of various frozen tissue samples, as physiological structures can vary across different sample spots.

**Figure 2.**
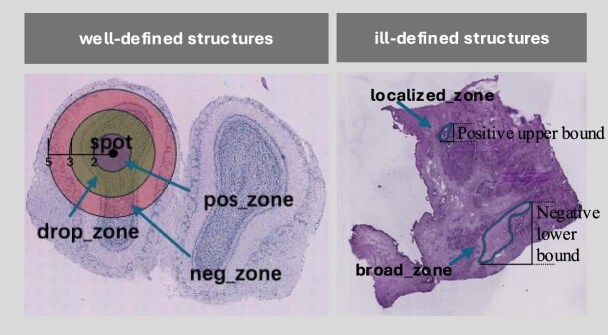
Adaptive positive and negative sample selection diagram. Left: in samples with well-defined structures (e.g. MOB), a spatially adjacent spot is selected as the positive sample for each anchor spot. To reduce ambiguity, spots at distances of 2–3 are excluded, while those at distances of 3–5 are selected as negative samples to ensure sufficient spatial separation. Right: in samples with ill-defined structures [e.g. pancreatic ductal adenocarcinoma (PDAC)], the sampling distance threshold for positive samples should be smaller than the size of the localized zone, while the sampling distance for negative samples should exceed the range of the broad zone. The positive upper bound is determined based on the size of the localized zone. Depending on the extent of the broad zone, the negative lower bound is further relaxed to enhance the spatial dissimilarity between positive and negative samples.

For instance, in the case of mouse MOB data (Fig. 2) which exhibit a ring-shaped hierarchical structure, the similarity among spots within each hierarchy tends to be higher. If we were to define positive sample spots solely based on immediate neighbors, this could inadvertently result in the inclusion of similar sites from the same physiological structure in the negative sample spots. To address this, we implement a triple concentric circle sampling strategy: the innermost circle represents the center of the positive sample spots, while the outermost circle encompasses the negative sample spots. This design aims to minimize the overlap of similar data across hierarchical layers.

In unsupervised settings, existing CL methods typically select negative samples at random, which may result in negative samples sharing the same label. Based on the approach proposed in this study, correcting for same-label bias among negative samples can further improve model performance in representation learning [44]; we recognize that the threshold for CL should be tailored to the specific characteristics of different spatial transcriptome datasets.

In samples with well-defined tissue structures, we manually define geometric regions (circles or rectangles) to select CL areas, including the positive zone (proximal region), drop zone (intermediate region), and negative zone (distant region), according to spatial distances. For instance, in the case of mouse MOB data with well-defined tissue structures, we define this threshold based on the size of a locus at the center of a region, establishing the radius for positive sample spots accordingly. Sample points in closer proximity to the target location are excluded to reduce the risk of misclassification. Points slightly further away, but still within a moderate range, are selected as negative samples to ensure adequate spatial separation.

In samples with ill-defined tissue structures, thresholds are determined based on the size of broad and localized regions. For localized regions, we set the upper bound of the positive zone threshold and the lower bound of the negative zone threshold. For broad regions, the upper bound of the negative zone threshold is further relaxed to accommodate structural ambiguity. The core adjustment strategy is to narrow the similarity threshold and increase the dissimilarity threshold.

To calculate the contrastive loss between the embeddings of positive and negative samples, assume we have a set of samples $\lbrace x_i\rbrace _{i=1}^N$, where each sample *x*_*i*_ has an embedding representation **e**_*i*_ and a corresponding position vector **p**_*i*_. The dynamic contrastive loss function $\mathcal {L}$ is defined as follows:


(1) \begin{eqnarray*}
\mathcal {L} = \frac{1}{N} \sum _{i=1}^N \left( \mathcal {L}_{\text{pos}}^i + \mathcal {L}_{\text{neg}}^i \right),
\end{eqnarray*}

where the positive sample loss $\mathcal {L}_{\text{pos}}^i$ is defined as:


(2) \begin{eqnarray*}
\mathcal {L}_{\text{pos}}^i = -\frac{1}{|P_i|} \sum _{j \in P_i} \log \frac{\exp (-\Vert \mathbf {e}_i - \mathbf {e}_j\Vert )}{\sum _{k \in P_i} \exp (-\Vert \mathbf {e}_i - \mathbf {e}_k\Vert )},
\end{eqnarray*}

The negative sample loss $\mathcal {L}_{\text{neg}}^i$ is defined as:


(3) \begin{eqnarray*}
\mathcal {L}_{\text{neg}}^i = -\frac{1}{|N_i|} \sum _{j \in N_i} \log \frac{\exp (\Vert \mathbf {e}_i - \mathbf {e}_j\Vert )}{\sum _{k \in N_i} \exp (\Vert \mathbf {e}_i - \mathbf {e}_k\Vert )},
\end{eqnarray*}

In the above formulas:

- *P*_*i*_ denotes the set of positive samples whose positional distance from sample *x*_*i*_ is less than the positive sample threshold recorded as threshold_pos and > 0;
- *N*_*i*_ denotes the set of negative samples whose positional distance from sample *x*_*i*_ is greater than the negative sample threshold recorded as threshold_neg and less than the radius threshold threshold_radius;
- ‖**e**_*i*_ − **e**_*j*_‖ represents the Euclidean distance between samples *x*_*i*_ and *x*_*j*_.

The positive sample loss $\mathcal {L}_{\text{pos}}^i$ is computed by weighting the embedding distances of the positive sample set *P*_*i*_, with closer sample pairs receiving higher weights. The negative sample loss $\mathcal {L}_{\text{neg}}^i$ is computed by weighting the embedding distances of the negative sample set *N*_*i*_, with further apart sample pairs receiving higher weights.

Through this dynamic contrastive loss function, the model can adaptively select positive and negative sample pairs based on their spatial positions, thereby improving the model’s effectiveness and robustness in contrastive learning.

#### High-generality model based on DANN

Real spatial transcriptome data and simulated spatial transcriptome data inherently differ, which can introduce biases in the performance of models trained on simulated data when applied to real data deconvolution. Domain adaptation addresses this issue by aligning the disparity between real spatial transcriptome data and simulated spatial transcriptome data, allowing the trained model to generalize more effectively on new data [45]; to mitigate this bias, we employ domain adversarial training. This technique facilitates transfer of learning between source and target domains [46], enabling models trained in the source domain to be effectively applied to data in the target domain. By using adversarial training, we reduce discrepancies between the distributions of the source and target domains and learn a generalized representation that enhances model performance in the target domain.

Our approach involves two key training strategies.

- We train the model on a substantial volume of simulated data (∼ 10 times more), utilizing CARD data for adversarial samples.
- We conduct fine-tuning after the initial training, specifically employing domain adversarial techniques to refine the model.

To quantify the similarity between the embeddings of simulated and real spatial transcriptome data, we calculate the maximum mean discrepancy (MMD) loss, as introduced by Gretton *et al.* [47], a widely used technique for measuring the discrepancy between distributions in the context of domain adaptation and feature alignment. MMD measures the distance between distributions *P* and *Q*, and is defined as follows:


(4) \begin{eqnarray*}
\text{MMD}(P, Q) = \Vert \mu _P - \mu _Q\Vert _{\mathcal {H}},
\end{eqnarray*}

where $\mu _P = \mathbb {E}_{x \sim P}[\phi (x)]$ and $\mu _Q = \mathbb {E}_{y \sim Q}[\phi (y)]$ are the mean embeddings of the distributions *P* and *Q* in the RKHS (reproducing kernel Hilbert space), respectively. The mapping ϕ(*x*) is induced by the kernel function *k*(*x*, *y*), which is crucial in determining the properties of the feature space. In this study, we use the Gaussian kernel function to compute the MMD loss. The Gaussian kernel function *k*(*x*, *y*) is defined as follows:


(5) \begin{eqnarray*}
k(x, y) = \exp \left(-\frac{\Vert x - y\Vert ^2}{2\sigma ^2}\right).
\end{eqnarray*}

The symbol σ represents the standard deviation of the Gaussian distribution. It controls the width of the Gaussian function and determines the sensitivity of the kernel function to the distance between input points *x* and *y*. In this study it is 1.0.

MMD is a non-parametric statistical method that effectively measures the difference between two probability distributions, making it particularly suitable for small sample sizes. Additionally, it exhibits robustness against outliers, which can skew results in parametric statistical methods. A small MMD value indicates that the mean embeddings of the two distributions are close in feature space, suggesting a high degree of similarity or equivalence between them. Conversely, a large MMD value indicates a significant difference between the two distributions, highlighting the need for further refinement in the model’s transferability from the source to the target domain.

The formula for computing the MMD loss is:


(6) \begin{eqnarray*}
\text{MMD}^2(P, Q) = \frac{1}{n^2} \sum _{i=1}^n \sum _{j=1}^n k(x_i, x_j) + \nonumber \\ \frac{1}{m^2} \sum _{i=1}^m \sum _{j=1}^m k(y_i, y_j) - \frac{2}{nm} \sum _{i=1}^n \sum _{j=1}^m k(x_i, y_j)
\end{eqnarray*}

- *P* and *Q* are the two distributions being compared, with *n* samples from *P* (positive sample spots) and *m* samples from *Q* (negative sample spots).
- *k*(*x*_*i*_, *x*_*j*_) represents the kernel function (in this case, often the Gaussian kernel) evaluated between two samples *x*_*i*_ and *x*_*j*_ from distribution *P*, or between two samples *y*_*i*_ and *y*_*j*_ from distribution *Q*, or between *x*_*i*_ from *P* and *y*_*j*_ from *Q*.
- The first term $\frac{1}{n^2} \sum _{i=1}^n \sum _{j=1}^n k(x_i, x_j)$ computes the mean pairwise similarity within samples from distribution *P*. The second term $\frac{1}{m^2} \sum _{i=1}^m \sum _{j=1}^m k(y_i, y_j)$ does the same for distribution *Q*.
- The third term $\frac{2}{nm} \sum _{i=1}^n \sum _{j=1}^m k(x_i, y_j)$ computes the mean similarity between samples from *P* and *Q*.

During the encoder training phase, we focus solely on optimizing the encoder’s ability to minimize the differences in embeddings, without adjusting the domain classifier. In contrast, during the domain classifier training phase, we fix the parameters of the encoder to enhance the discriminatory power of the domain classifier. We refer to this sequence of training—encoder training followed by domain classifier training—as one round, and we establish the number of rounds at 50 based on the marginal benefits observed from adversarial training. Notably, when the simulated spatial transcriptomics data lack biological realism and exhibit high randomness, the DANN module may negatively impact the prediction results. In such cases, it may be advisable to disable the DANN component to avoid performance degradation caused by domain mismatch.

### Prediction module

In this study, the prediction module is tasked with transforming the processed feature vectors into the final output predictions. This module receives high-dimensional embeddings generated by the upstream encoder module and processes them through a series of neural network layers. Initially, the embeddings are passed through a linear layer followed by a leaky ReLU activation function, which introduces non-linearity. This is subsequently followed by a dropout operation to mitigate overfitting, reducing the dimensionality to 128. The resulting output is then fed into another linear layer, culminating in the application of a softmax function to generate the model’s prediction output. The primary objective of this module is to accurately predict cell type proportions based on the input features.

(i) Input layer: this layer receives high-dimensional embeddings from the encoder, with an input dimension of *d*_input_.

(ii) Hidden layer: the input dimension is initially reduced to 128 through a linear transformation, facilitating a more compact representation of the data. A leaky ReLU activation function is then applied to enhance the model’s ability to capture non-linear relationships within the data. To mitigate overfitting, dropout is employed, retaining 50% of the nodes during training.

(iii) Output layer: the final transformation of features is executed through a linear layer, followed by the application of a softmax function to produce a probability distribution for the predicted cell types.

Specifically, the architecture of the prediction module can be expressed as:


(7) \begin{eqnarray*}
\mathbf {y} = \text{softmax}\left( \mathbf {W}_2 \cdot \text{Dropout}\left( \text{Leaky ReLU}\left( \mathbf {W}_1 \cdot \mathbf {x} + \mathbf {b}_1 \right) \right) + \mathbf {b}_2 \right)\nonumber\\
\end{eqnarray*}

where **W**_1_ and **W**_2_ are weight matrices, **b**_1_ and **b**_2_ are bias terms, **x** is the input feature vector, and **y** is the final prediction output (cell type probability distribution).

This architectural design allows the prediction module to effectively capture the relationship between the input features and the target variable, which is the cell type proportions, thus enabling accurate predictions of cell types. To optimize the parameters of both the encoder and prediction modules, we employed the Adam optimizer. Specifically, we combined the parameters from both modules for joint optimization using the Adam optimizer, with the learning rate set according to the value specified in the training parameters. In addition, the configuration of the optimizer is presented in Eq. (8) as a code snippet. The configuration of the optimizer is as follows:


(8) \begin{eqnarray*}
\mathrm{optimizer\_predictor} = \text{Adam}\big (\text{list}(\text{encoder.parameters()}) +\nonumber \\ \text{list}(\text{predictor.parameters()}), \text{lr} = \mathrm{learning\_rate} \big )\nonumber\\
\end{eqnarray*}

Here, Adam is an adaptive learning rate optimization algorithm that effectively handles sparse gradients and has demonstrated excellent convergence speed and accuracy in many tasks. By employing this approach, we ensure that the encoder and prediction modules in the model are adequately optimized during training, thereby improving the overall model performance.

The deconvolution results are clustered into the same number of clusters as the cell types. To evaluate the effectiveness of our predictions, we employ performance metrics specifically designed for clustering validation. The adjusted Rand index (ARI) is a widely used metric to assess the similarity between two data clusterings, with adjustments made for random agreements. The ARI is calculated as follows:


(9) \begin{eqnarray*}
\text{ARI} = \frac{\sum _{i=1}^{k} \sum _{j=1}^{l} \binom{n_{ij}}{2} - \frac{\sum _{i=1}^{k} \binom{a_i}{2} \sum _{j=1}^{l} \binom{b_j}{2}}{\binom{n}{2}}}{\frac{1}{2} \left( \sum _{i=1}^{k} \binom{a_i}{2} + \sum _{j=1}^{l} \binom{b_j}{2} \right) - \frac{\sum _{i=1}^{k} \binom{a_i}{2} \sum _{j=1}^{l} \binom{b_j}{2}}{\binom{n}{2}}}
\end{eqnarray*}

where:

- *n*: total number of samples,
- *k* and *l*: number of true classes and predicted clusters, respectively,
- *n*_*ij*_: number of samples shared by true class *C*_*i*_ and predicted cluster *K*_*j*_,
- *a*_*i*_: number of samples in true class *C*_*i*_, and
- *b*_*j*_: number of samples in predicted cluster *K*_*j*_.

The ARI ranges from –1 to 1, where 1 indicates a perfect match between clustering and true labels, 0 indicates clustering no better than random, and values below 0 suggest clustering that is less accurate than random assignment.

Purity, a simpler yet effective metric, evaluates how well each cluster corresponds to a single true class. It is defined as:


(10) \begin{eqnarray*}
\text{Purity} = \frac{1}{n} \sum _{j=1}^{l} \max _{i} |C_i \cap K_j|
\end{eqnarray*}

where:

- *n*: total number of samples,
- *C*_*i*_: the *i*th true class,
- *K*_*j*_: the *j*th predicted cluster, and
- |*C*_*i*_∩*K*_*j*_|: number of samples shared by true class *C*_*i*_ and predicted cluster *K*_*j*_.

Purity ranges from 0 to 1, with higher values indicating stronger alignment between clusters and true classes. A purity of 1 signifies that each cluster contains only samples from a single true class, reflecting ideal clustering performance.

To quantitatively evaluate the consistency between the predicted cell type proportions and the ground truth labels, we employed two widely used metrics: the Jensen–-Shannon (JS) divergence and the Pearson correlation coefficient.

Given the predicted vector **p** and the ground truth vector **q** for each spatial location, we first normalize both vectors to ensure they form valid probability distributions. The JS divergence is then calculated as:


(11) \begin{eqnarray*}
\mathrm{JS}(\mathbf {p}, \mathbf {q}) = \frac{1}{2} D_{\mathrm{KL}}(\mathbf {p} \parallel \mathbf {m}) + \frac{1}{2} D_{\mathrm{KL}}(\mathbf {q} \parallel \mathbf {m}), \quad \text{where } \mathbf {m} = \frac{1}{2} (\mathbf {p} + \mathbf {q})\nonumber\\
\end{eqnarray*}

where *D*_KL_ denotes the Kullback–Leibler divergence. A smaller JS divergence indicates a higher similarity between the predicted and true distributions.

To capture the linear relationship between predicted and true proportions, we also compute the Pearson correlation coefficient, which is defined as:


(12) \begin{eqnarray*}
\mathrm{Corr}(\mathbf {p}, \mathbf {q}) = \frac{\sum _{i=1}^{n}(p_i - \bar{p})(q_i - \bar{q})}{\sqrt{\sum _{i=1}^{n}(p_i - \bar{p})^2} \sqrt{\sum _{i=1}^{n}(q_i - \bar{q})^2}}
\end{eqnarray*}

where $\bar{p}$ and $\bar{q}$ denote the mean values of vectors **p** and **q**, respectively. A higher correlation indicates better alignment between predicted and ground truth distributions.

The final evaluation results are obtained by averaging both metrics across all spatial locations.

## Results

### Datasets

Our study evaluated the performance of ST-deconv using three types of datasets: CARD-simulated spatial transcriptomics data, ST-deconv-simulated spatial transcriptomics data, and real MOB spatial transcriptomics data.

#### CARD and ST-deconv simulate spatial transcriptomics data

In this study, we utilized MOB scRNA-seq data consisting of 12 801 cells and 18 560 genes as a reference to guide spatial transcriptomics analysis. Simulated MOB spatial transcriptomics data generated by CARD were used, categorized into five groups (Data IDs 01–05) with strong spatial correlation and five groups (Data IDs 31–35) with weaker spatial correlation. The entire dataset includes 260 cells and 18 263 genes. During the experiments with simulated data, ST-deconv generated larger datasets, equivalent to 10 times the number of real spatial transcriptomics spots, e.g. totaling 2820 MOB spots—to facilitate large-scale training.

#### Real mouse olfactory bulb spatial transcriptomics data

The MOB [17] is a critical brain region responsible for processing olfactory information. The MOB’s structure is composed of several distinct layers, each involving different types of neurons that contribute to olfactory signal processing. These layers include:

- Olfactory nerve layer (ONL): this layer consists of axons from olfactory sensory neurons, responsible for transmitting odor signals from the nasal cavity to the olfactory bulb.
- Glomerular layer (GL): this is the site where olfactory sensory neurons form synaptic connections with secondary neurons, such as tufted and mitral cells, facilitating the initial relay of olfactory information.
- External plexiform layer (EPL): containing tufted cells and various interneurons, the EPL plays a crucial role in modulating olfactory signals.
- Granule cell layer (GCL): composed of inhibitory interneurons called granule cells, this layer enhances signal contrast by regulating the activity of mitral cells.

In our study, we utilized MOB data as real spatial transcriptome data to validate our model’s performance. By comparing model-predicted expression values with observed data across different layers and corresponding cell types, we assessed the accuracy of the model in capturing the complex spatial organization of gene expression in the MOB.

#### Pancreatic ductal adenocarcinoma spatial transcriptomics data

We performed deconvolution analysis using scRNA-seq data from pancreatic ductal adenocarcinoma (PDAC) and its corresponding spatial transcriptomics data [48]. The scRNA-seq dataset contains 1925 cells, while the spatial transcriptomics dataset comprises 428 spatial spots. Due to the limited number of spatial spots, all gene dimensions were retained without applying gene selection. Given the weak structural definition, the simulated data generated for training may not accurately reflect the biological characteristics of the real tissue. To mitigate potential overfitting or training instability caused by domain adversarial learning on such less realistic data, the DANN module was temporarily excluded from the deconvolution process for PDAC, ensuring more stable and interpretable predictions. To further support the spatial validity of the deconvolution results in the absence of clear structural boundaries, we incorporated the spatial expression patterns of representative marker genes as a reference for evaluating the predicted cell type distributions. Specifically, we selected four representative cell types along with their corresponding marker genes: Cancer_clone_A (TM4SF1), Ductal_MHC_Class_II cells (C4A), Ductal_CRISP3_highcentroacinar_like cells (CRISP3), and Fibroblasts (CD248). Furthermore, we quantitatively compared the proportion of each cell type in the scRNA-seq data and in the deconvolution results, aiming to evaluate the model’s generalization capability on tissue samples with ambiguous spatial structure.

### Optimizing module for enhanced model performance

To evaluate the impact of each module on model performance, we assessed the model with various module combinations, using root mean square error (RMSE) as the primary metric. RMSE serves as a critical measure of prediction accuracy, with lower values reflecting reduced prediction errors. Table 1 provides a summary of the experimental results for each module combination.

**Table 1.** Ablation experiment results

ST-deconv	Ablation	Gene filter	RMSE	CARD simu data	Weighted
simu	Experiment	*t*-test	Min	RMSE	value
0	Base_model+CL+DANN	$\checkmark$	0.0502	0.0700	0.0551
0	Base_model+CL+DAN		0.0507	0.0759	0.0570
0	Base_model	$\checkmark$	0.0521	0.0738	0.0576
0	Base_model+CL	$\checkmark$	0.0522	0.0750	0.0579
0	Base_model		0.0527	0.0748	0.0582
0	Base_model+DANN	$\checkmark$	0.0519	0.0779	0.0584
0	Base_model+CL		0.0531	0.0759	0.0588
0	Base_model+DANN		0.0526	0.0888	0.0616
1	Base_model+simu+CL		0.0118	0.2439	0.0745
1	Base_model+simu+CL	$\checkmark$	0.0124	0.2745	0.0775
1	Base_model+simu	$\checkmark$	0.0136	0.2791	0.0792
1	Base_model+simu+DANN		0.0185	0.2833	0.0801
1	Base_model+simu+DANN	$\checkmark$	0.0177	0.2855	0.0846
1	Base_model+simu		0.0180	0.3255	0.0953
1	Base_model+simu+CL+DANN	$\checkmark$	0.0181	0.3288	0.0958

The table presents the RMSE and weighted values obtained from different ablation experiments. When using ST-deconv to simulate spatial transcriptome data, ST-deconv simu is set to 1; otherwise, it is set to 0. Both CARD and ST-deconv simulated data are split into training and test sets. The minimum RMSE (RMSE Min) is the evaluation result on the test data, while the CARD-simulated data (Data ID 04 RMSE) represents a different set of simulated data from the split data. The weight value is calculated as a weighted average of RMSE Min and CARD simu Data ID 04 RMSE to minimize the overall value range across different ablation experiments.

Base_model represents the most streamlined model configuration, including only the pre-processing, encoder, and predictor components.

As shown in Table 1, applying only the encoder and DANN modules yields a minimum RMSE of 0.0526, a substantial reduction compared with the standalone encoder model. For all other combinations in the ablation study, RMSE values remain consistently lower when the DANN module is included, highlighting DANN’s effectiveness in bridging distributional differences between the source and target domains, and enhancing model generalization. However, when tested on data generated through ST-deconv random simulation, the integration of the DANN module did not lead to performance improvement. Although the random simulation method provides more accurate composition ratios of spatial locations and cell types, its high randomness introduces larger distributional gaps compared with real spatial transcriptomics data. In this case, the DANN module’s strong discriminative power may have caused overfitting to the simulated data distribution, resulting in slightly worse prediction outcomes than the configuration without the DANN module.

Incorporating the CL module further reduces the minimum RMSE for the Base_model + DANN model from 0.0526 to 0.0507, underscoring CL’s impact in improving feature representation and reducing category confusion. Notably, the combination of DANN and CL modules achieves the most significant improvement, with a minimum RMSE of 0.0507, which emphasizes the substantial performance gains through module synergy.

Including the *t*-test module yields a slight improvement, reducing the minimum Base_model + CL + DANN model from 0.0507 to 0.0502.

Overall, the lowest RMSE is attained with the full module combination of Base_model + DANN + CL + *t*-test, underscoring the importance of module integration in optimizing model performance. These results suggest that combining domain adaptation, CL, and statistical validation robustly enhances the model’s predictive capacity within the target domain.

### ST-deconv performance evaluation

**Figure 3.**
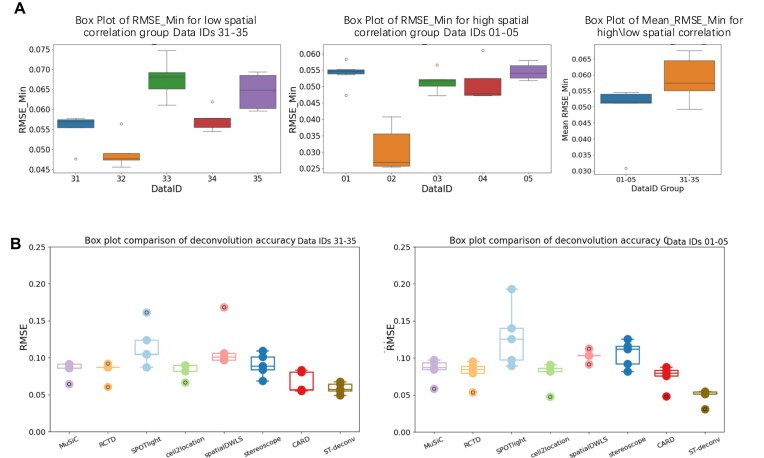
Comparative analysis using CARD-generated simulated MOB spatial transcriptomics data. (**A**) ST-deconv performance: to evaluate model stability, we conducted 5-fold cross-validation based on spatial transcriptomics data with varying levels of spatial correlation. The box plot on the far right visually highlights the influence of spatial information correlation on average RMSE values. (**B**) Deconvolution method comparison: this panel compares the RMSE of various deconvolution methods applied to spatial transcriptomics data with varying levels of spatial correlation.

To evaluate the model’s stability and generalization across different datasets, we used CARD-generated simulated MOB spatial transcriptomics data and applied 5-fold cross-validation to each dataset. The results are presented in the form of box plots, as shown in Fig. 3A. We evaluated the distribution of RMSE minima for two different groups of datasets (Data IDs 01–05 and Data IDs 31–35) using 5-fold cross-validation for each. By comparing the RMSE distributions of these two dataset groups, we can observe the impact of the differences in spatial information between them on the model performance. Both datasets are derived from simulated spatial transcriptome data from the CARD release MOB, with Data IDs 01–05 having higher spatial correlation settings at simulation and Data IDs 31–35 having lower spatial correlation.

Dataset 01–05 intuitively contains more spatial information, with cells showing more pronounced spatial clustering and organization patterns. The figure shows that dataset 02 has the lowest median minimum RMSE of ∼0.03 in this dataset, indicating that the model performs best on this dataset and is able to capture spatial dependence effectively. Comparatively, datasets Data IDs 01, 03, and 05 have a higher median minimum RMSE of ∼0.05–0.055, indicating that the model has a slightly larger error on these datasets. In contrast, dataset Data ID 32 also has a lower median minimum RMSE of ∼0.03, and the model performs better on this dataset. Datasets Data IDs 33 and 35 have higher medians (∼0.05–0.055) and more fluctuating RMSE values, suggesting that the model’s performance on these datasets is not as stable as on dataset Data ID 32. The higher median and wider distribution range are consistent with the fact that Data IDs 31–35 datasets contain less spatial information, and the average of minimum RMSE box plots also show that the model’s performance is better for the more spatial Data IDs 01–05 datasets. In conclusion, although the model’s performance varies in different datasets, the model maintains good stability in most of the datasets, which is also consistent with our understanding of spatial information stability, and is consistent with our intuition about the ability of spatial information to guide the existence of model deconvolution.

To further evaluate the stability and generalization capability of the model, we calculated the JS divergence and Pearson correlation coefficient for each fold during 5-fold cross-validation. After training on the five folds of the Data ID 02 dataset, the models obtained from each fold were independently validated on the Data ID 32 dataset. The results consistently showed strong performance, further demonstrating the model’s robustness and transferability across different datasets (Table 2).

### Comparison of ST-deconv with other methods

In this study, we used CARD-simulated datasets (high spatial correlation scenarios, Data IDs 01–05; low spatial correlation scenarios, Data IDs 31–35) generated from the same single-cell transcriptomics data and calculated the RMSE between the estimated and true cell type compositions for each method to evaluate their deconvolution performance. The RMSE values for the two sets of simulated data are displayed in the form of box plots in Fig. 3B.

From the plots, it can be observed that ST-deconv consistently exhibits low RMSE medians in both datasets (high/low spatial correlation scenarios, Data IDs 01–05 and 31–35) with narrow box plots, indicating high accuracy and stability. In the high spatial correlation scenario represented by Data IDs 01–05, ST-deconv achieves a lower RMSE median compared with most other methods, indicating smaller deconvolution errors and higher precision. ST-deconv not only shows a lower median but also a significantly narrower box plot, suggesting ST-deconv’s high consistency with minimal performance variability across different simulation conditions.

Additionally, ST-deconv has almost no outliers, further demonstrating its robustness, as it maintains stable deconvolution accuracy across multiple simulations without extreme deviations. This consistency is particularly valuable in biological research, where the stability and reproducibility of results are crucial.

Under low spatial correlation conditions represented by Data IDs 31–35, ST-deconv still maintains a relatively low RMSE median and a narrow box plot, exhibiting high accuracy and stability under this challenging condition. This robustness suggests that ST-deconv has low error variability and good adaptability to spatial noise.

Overall, the box plot analysis highlights that ST-deconv outperforms other methods in terms of accuracy (low RMSE median) and stability (narrow box plot with few outliers). These results underscore ST-deconv’s adaptability, making it suitable for a range of spatial transcriptomics data conditions, from highly structured environments to noisier, low spatial correlation data, extending its applicability across different spatial contexts.

### Application of ST-deconv to real and simulated spatial transcriptomics data

In this study, ST-deconv integrates spatial information and cell type characteristics to accurately infer cell type composition at each spatial locus from low-resolution spatial transcriptome data. It offers the flexibility to either autonomously generate or directly input existing spatial transcriptome datasets. After training with ST-deconv-simulated spatial transcriptome data, ST-deconv was successfully applied to deconvolve real spatial transcriptome data, effectively inferring cell type proportions and generating spatial distribution maps.

**Figure 4.**
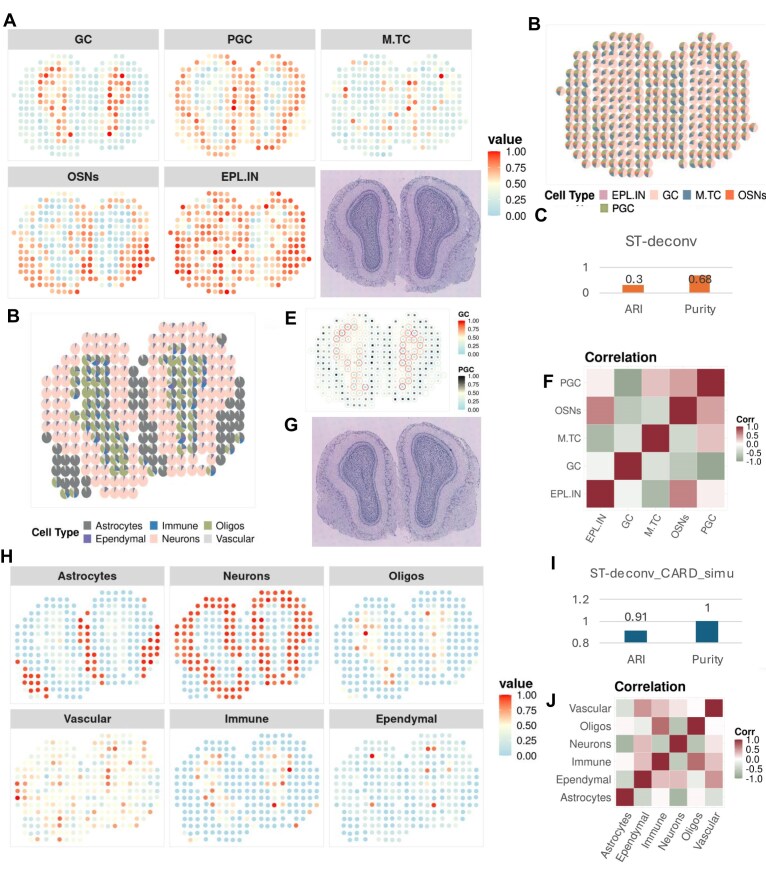
Analysis results based on real and CARD-simulated spatial transcriptomic data. (**A**) Cell type distribution in deconvolution: display of post-deconvolution cell type distributions in MOB spatial transcriptomic data, illustrating hierarchical structures and specimen-specific patterns. (**B**) Cell type maps in deconvolution: spatial distribution of selected cell types [external plexiform layer interneurons (EPLEPL.INs), granular cells (GCs), mitral/tufted cells (M.TCs), olfactory sensory neurons (OSNs), and periglomular cells (PGCs)] in MOB data, emphasizing spatial relationships derived from deconvolution. (**C**) Deconvolution performance: assessment of deconvolution accuracy in MOB spatial transcriptome data using ARI and purity metrics, with specimen layer labels as a reference. (**D**) Cell type differentiation in CARD-simulated data: cell type distribution percentages specific to Data ID 04.(**E**) Detailed comparison of GCs and PGCs: focused schematic comparing GCs and PGCs in real MOB data to showcase differentiation, (**F**) Cell type correlation map: correlation of spatial relationships among various cell types within the MOB dataset. (**G**) Hierarchical differentiation in layers: prominent hierarchical layer differentiation in CARD-simulated MOB spatial transcriptome data. (**H**) Cell type distribution in CARD-simulated data: spatial distribution of specific cell types (astrocytes, neurons, oligos, vascular, immune, and ependymal) in CARD-simulated MOB data, reflecting inferred spatial relationships from deconvolution. (**I**) Deconvolution performance in CARD-simulated data: evaluation of deconvolution performance for Data ID 04 using ARI and purity metrics, referencing specimen layer labels. (**J**) Correlation of cell types in simulated data: corresponding specimen diagram for simulated data in Data ID 04, highlighting cell type association.

To assess the accuracy of the ST-deconv model, we compared its deconvolution results with known tissue structures, particularly using MOB data (Fig. 4A), which display distinct circular hierarchical organization. The analysis confirmed that the spatial distribution of cell types in the deconvolution results aligned with biological expectations, accurately capturing the hierarchical structure. Figure 4C presents the overall cell type deconvolution for the real MOB dataset.

Figure 4C displays deconvolution results following clustering assessed by the ARI, with annotated labels for the GCL, GL, mitral cell layer (MCL), and ONL. The purity metric achieved a value of 0.68. To calculate ARI and purity, the deconvolution results are clustered into the same number of clusters as the cell types, then compared with the hierarchical annotation information of the original specimen structure. These findings demonstrate the robust performance of ST-deconv in the deconvolution of real spatial transcriptome data. To facilitate a more detailed comparison of deconvolution performance among various methods, the ARI and purity were calculated for various spatial transcriptomics deconvolution methods, as summarized in Table 3. Among the compared methods, SpatialDWLS achieved the highest ARI score of 0.44, indicating strong consistency with ground truth annotations, while ST-deconv demonstrated competitive performance with an ARI of 0.30 and the highest purity score of 0.68, reflecting accurate identification of dominant cell types. CARD also performed well across both metrics. These results highlight the effectiveness of ST-deconv in preserving cell type purity while maintaining clustering consistency.

**Table 2.** Five-fold cross-validation between Data ID 02 (train) and Data ID 32 (test), evaluated by JS divergence and Pearson correlation

Fold	JS divergence	Pearson correlation
fold0	0.2162	0.9317
fold1	0.2009	0.8956
fold2	0.2086	0.9296
fold3	0.2033	0.9414
fold4	0.2131	0.8575
**Average**	**0.2088**	**0.9111**

**Table 3.** Comparison of ARI and purity scores across different spatial deconvolution methods

Method	ARI	Purity
ST-deconv	0.30	0.68
MuSiC	0.30	0.52
RCTD	0.26	0.58
SPOTlight	0.29	0.53
Cell2location	0.25	0.49
SpatialDWLS	0.44	0.63
Stereoscope	0.28	0.55
CARD	0.40	0.66

In addition to real spatial transcriptome data, we also conducted experiments using CARD-simulated spatial transcriptome data (high spatial correlation scenarios, Data ID 04) to assess ST-deconv’s adaptability to varying spatial correlations. Figure 4D, G, and H show deconvolution results for specific cell types in simulated spatial transcriptome data, highlighting spatial relationships and hierarchical distributions characteristic of the MOB dataset. Figure 4F presents a correlation heatmap showcasing the spatial distribution of various cell types in the olfactory bulb, highlighting either synergistic or independent distributions. Cells closely associated with olfactory sensory neurons (OSNs), such as periglomular cells (PGCs) and mitral/tufted cells (M.TCs), exhibited a strong positive correlation, underscoring their co-existence in olfactory signal processing.

In our examination of granular cells (GCs) and PGCs, we noted their weak correlation, as illustrated in Fig. 4E. GCs, which are inhibitory neurons in the olfactory bulb, are predominantly found in the deep layers, with the red area in the figure indicating their dense distribution. Conversely, PGCs are located near the GL and play a crucial role in regulating signals from OSNs. The concentration map of PGCs illustrates their clustering in specific glomerular regions, consistent with their function in initial signal processing within the olfactory bulb. Inhibitory cells, such as PGCs and GCs, displayed high concentrations in localized regions, reflecting their regulatory roles. OSNs, however, were more diffusely distributed, indicative of their function as input neurons. The distribution of M.TCs and external plexiform layer interneurons (EPL-INs) further corresponded with their roles in olfactory signaling and regulation.

Figure 4I displays deconvolution results following clustering assessed by the ARI, with annotated labels for six cell types. The purity metric achieved a value of 1. These findings demonstrate the robust performance of ST-deconv in the deconvolution of CARD-simulated spatial transcriptome data. Figure 4J presents a correlation heatmap showcasing the spatial distribution of various cell types. These findings highlight the strong performance of ST-deconv in accurately deconvoluting spatial transcriptomic data.

**Figure 5.**
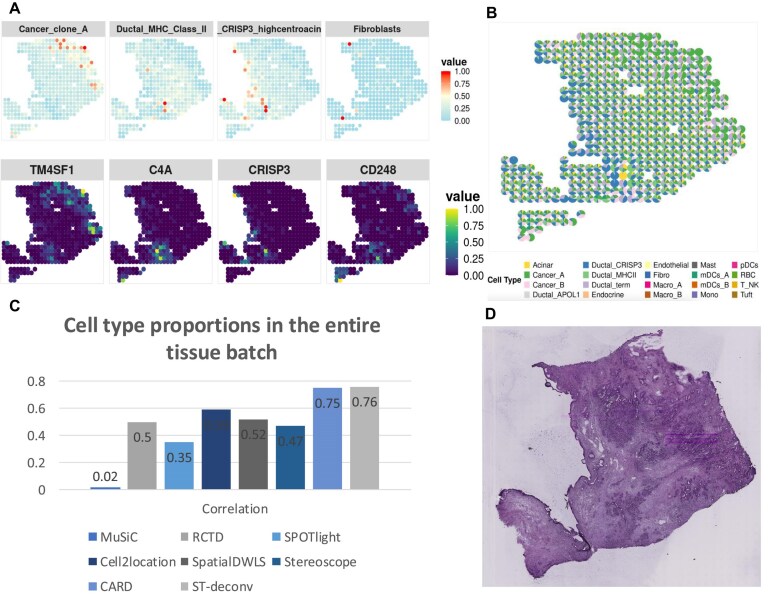
Analysis results based on real PDAC spatial transcriptomics data. (**A**) Partial cell type distribution and corresponding marker gene expression in PDAC: visualization of representative cell types after deconvolution and the spatial expression patterns of their corresponding marker genes. (**B**) Spatial distribution of all cell types in the PDAC sample: visualization of deconvolution results for all cell types across the full PDAC tissue section. (**C**) Overall cell type proportions and correlations in PDAC: comparison of cell type proportions between scRNA-seq data and deconvolution results, along with their correlation analysis. (**D**) Histological image of the frozen PDAC tissue section: the original pathology image of the real PDAC sample, used as a spatial reference.

In addition to the analysis on the well-defined tissue structure of the MOB dataset, we also conducted deconvolution experiments on real spatial transcriptomics data from a PDAC tissue sample characterized by ill-defined tissue structures. Figure 5 presents the results of this experiment, showing the spatial distributions of several representative cell types obtained through deconvolution, alongside the spatial expression patterns of their corresponding marker genes, highlighting the spatial concordance between the predicted cell types and gene expression signals. Figure 5B illustrates the spatial distribution of all cell types across the entire PDAC tissue section, revealing cellular heterogeneity. Figure 5C quantitatively compares the cell type proportions inferred from the deconvolution results of different methods with those derived from the scRNA-seq data, and further evaluates the correlations between them. Figure 5D presents the histological image of the frozen PDAC tissue section, serving as a morphological reference for spatial interpretation.

## Discussion

In this study, we developed and validated the ST-deconv model, an inverse convolutional framework specifically designed for processing spatial transcriptome data. By training on simulated spatial transcriptome data and applying the model to real datasets, we demonstrated that ST-deconv effectively parses complex spatial transcriptome data and accurately infers the cell type composition of each spatial locus. Notably, the model successfully reproduced biologically relevant cell type distributions in samples exhibiting clear spatial hierarchical structures, such as the MOB. A correlation heatmap indicated a strong positive correlation (deep red) between EPL-INs and OSNs, suggesting an interesting biological relationship. Although EPL-INs are typically located in the external plexiform layer while OSNs project to the glomerular layer, this positive correlation may indicate a functional spatial interplay, particularly in olfactory signal processing, where EPL-INs may modulate excitatory signals from OSNs. Previous research has successfully used spatial transcriptomics to analyze the mechanisms of MOB [49]. Thus, this study provides valuable insights into the biological implications of spatial transcriptomics.

In the PDAC dataset, although no clear anatomical partition is observed at the physiological level, we manually inspected the spatial features extracted by the model and selected appropriate regions for positive zones, drop zones, and negative zones within the CL module. Based on this configuration, the correlation between the predicted cell type proportions and those derived from single-cell transcriptomics of the same tissue remained at a competitive level compared with similar methods, demonstrating the versatility of the CL module across different data types. Furthermore, the spatial comparison between predicted cell types and the expression patterns of their marker genes further validated the accuracy of the deconvolution results.

The ST-deconv model was meticulously designed to integrate spatial information and cell type characteristics. While spatial transcriptome data inherently possess spatial context, incorporating this information for effective deconvolution presents a key challenge. Inspired by the observation that proximate spatial locations exhibit similar gene expression patterns, we introduced CL into our model. This approach utilizes positive and negative loss to compute differences between locus pairs, thereby optimizing training to effectively handle biologically relevant spatial dependencies. To enhance the model’s ability to deconvolve real data, we incorporated DANN, which improved the model’s generalization capabilities.

The primary advantages of the ST-deconv model over existing deconvolution methods include the following. (i) High accuracy: by leveraging spatial information, ST-deconv resolves cell types more precisely, especially in structurally complex tissues. (ii) Flexibility: the model’s design allows for the integration of DANN, facilitating its application to various types of spatial transcriptome data, including both high-noise (simulated) and low-resolution datasets. (iii) Scalability: ST-deconv is adaptable and can be trained on multiple sources of data, whether generated autonomously or derived from direct input.

Despite its strong performance, the ST-deconv model has certain limitations. (i) Sensitivity to high-noise data: while the model excels with low-resolution data, since ST-deconv relies on DANN to adversarially learn the distribution differences betwenn simulated and real data, the quality of the simulated data has a direct impact on the model's final performance. Its deconvolution performance diminishes with high-noise inputs, such as lower fidelity simulated spatial transcriptome data. Improved simulated datasets may mitigate this issue, and advancements in spatial transcriptomics technology could enable the use of real single-cell spatial transcriptomes for training the model. (ii) Computational complexity: the integration of spatial information increases the model’s computational complexity, potentially resulting in longer processing times and higher resource demands when applied to large-scale datasets.

Our experimental results indicate that the ST-deconv model accurately captures spatial structure and cell type distribution in biological tissues, providing a novel tool for investigating cellular interactions and advancing spatial transcriptomics research. In particular, the model effectively reproduced the known circular hierarchical structure in MOB data, validating its efficacy and highlighting its potential for application in complex biological samples.

Future studies can further optimize and extend the ST-deconv model in several key areas. (i) Model optimization: enhancements can be made to our model’s design for simulating spatial transcriptome data. Incorporating cell frozen sample photographs in data simulation may yield higher quality spatial transcriptome data, thereby improving the model’s deconvolution capabilities for low-resolution samples. (ii) Multimodal data fusion: we aim to explore the integration of ST-deconv with other data types, such as scRNA-seq and proteomic data, to enrich our understanding of tissue structure and function. (iii) Extensive validation: further validation of the model’s generalizability across diverse biological contexts will be essential for establishing its robustness and applicability in varied research scenarios.

## Conclusion

This study introduces the ST-deconv model, a novel inverse convolutional framework designed specifically for spatial transcriptome data deconvolution. Through comprehensive training on simulated data and rigorous application to real datasets, ST-deconv demonstrates the ability to accurately infer cell type compositions and capture biologically relevant spatial structures. The model’s integration of spatial information and its flexibility in handling both high-noise and low-resolution data offer distinct advantages over existing methods. Despite the high computational complexity and the sensitivity of the DANN module to poorly simulated data, ST-deconv still performs satisfactorily, particularly in structurally complex tissues such as the MOB. The results of 5-fold validation for JS divergence and Pearson correlation indicate that the predicted cell type proportion distributions are relatively close to the ground truth, with strong linear correlation. In the deconvolution results of the PDAC dataset compared with single-cell data, the sample-wise correlation of cell type proportions outperforms existing methods. On the MOB dataset, the model also shows clear advantages in key performance metrics such as RMSE and purity, further demonstrating its robustness and generalization ability across different tissue types. Future improvements, such as enhanced data simulation techniques, multimodal data integration, and broader validation, hold the potential to further optimize the model and extend its application to diverse biological contexts. In particular, leveraging biological information from real frozen tissue images to enhance data simulation capabilities can better utilize the strengths of the DANN module, allowing the model to automatically determine suitable contrastive sampling thresholds from the sample images, making it more user-friendly. This work presents an important contribution to spatial transcriptomics research, particularly by innovatively introducing spatial information through contrastive learning. It provides a novel tool and perspective for investigating cellular interactions within spatially organized tissues.

## Data Availability

All Python codes and datasets associated with this study have been permanently archived in Zenodo (https://doi.org/10.5281/zenodo.16019345) and are also publicly accessible on GitHub (https://github.com/julieDai/ST-deconv-analyze.git). The raw MOB spatial transcriptomics data are available at: https://www.spatialresearch.org/resources-published-datasets/doi-10-1126science-aaf2403/. The raw data for the PDAC dataset can be accessed via the NCBI Gene Expression Omnibus (GEO) under accession number: https://www.ncbi.nlm.nih.gov/geo/query/acc.cgi?acc=GSE111672.
